# The Digital Engagement of Older People: Systematic Scoping Review Protocol

**DOI:** 10.2196/25616

**Published:** 2021-07-05

**Authors:** Abraham Sahilemichael Kebede, Lise-Lotte Ozolins, Hanna Holst, Kathleen Galvin

**Affiliations:** 1 School of Health Sciences University of Brighton Brighton United Kingdom; 2 Department of Health and Caring Sciences Linnaeus University Växjö Sweden

**Keywords:** digital divide, digital engagement, digital inclusion, initial adoption, older people, older users, sustained engagement, technological nonuse, older adults

## Abstract

**Background:**

There is an ongoing negative narrative about aging that portrays older people as a socioeconomic burden on society. However, increased longevity and good health will allow older adults to contribute meaningfully to society and maximize their well-being. As such, a paradigm shift toward healthy and successful aging can be potentially facilitated by the growing digital technology use for mainstream (day-to-day activities) and assisted living (health and social care). Despite the rising digital engagement trend, digital inequality between the age groups persists.

**Objective:**

The aims of this scoping review are to identify the extent and breadth of existing literature of older people’s perspectives on digital engagement and summarize the barriers and facilitators for technological nonuse, initial adoption, and sustained digital technology engagement.

**Methods:**

This review will be based on the Arksey and O’Malley framework for scoping reviews. The 6-stage framework includes: identifying research questions, identifying relevant studies, study selection, charting the data, summarizing and reporting the results, and a consultation exercise. Published literature will be searched on primary electronic databases such as the Association of Computing Machinery, Web of Science, MEDLINE, PsycINFO, CINAHL, and ScienceDirect. Common grey literature sources will complement the database search on the topic. A two-stage (title/abstract and full article) screening will be conducted to obtain eligible studies for final inclusion. A standardized data extraction tool will be used to extract variables such as the profile of the study population, technologies under investigation, stage of digital engagement, and the barriers and facilitators. Identified and eligible studies will be analyzed using a quantitative (ie, frequency analysis) and qualitative (ie, content analysis) approach suitable for comparing and evaluating literature to provide an evaluation of the current state of the older person’s digital engagement. Inclusion will be based on the Joanna Briggs Institute–recommended participant, concept, and context framework. Articles on older people (65 years and older), on digital technology engagement, and from a global context will be included in our review.

**Results:**

The results of this review are expected in July 2021.

**Conclusions:**

The findings from this review will identify the extent and nature of empirical evidence on how older people digitally engage and the associated barriers and facilitators.

**International Registered Report Identifier (IRRID):**

PRR1-10.2196/25616

## Introduction

### Background

Global demographic trends show that the worldwide age structure is rapidly changing more than ever before. The United Nations defines older people as those aged 65 years or older based on people’s chronological age. Currently, there are over 703 million older people, and it is expected to reach 2.1 billion by the year 2050 [1,2]. Population projections have indicated Europe and North America have the fastest growing aging population, and by 2050, the population percentage of older adults is expected to reach 34% in Europe and 28% in North America [3].

There is an ongoing negative narrative about aging that age-related changes, disability, and dependency among older people with poor and deteriorating health conditions imply an increased expenditure on health and its burden on the socioeconomic aspects of society [4]. Further, the COVID-19 pandemic has also underlined how older people are generally perceived and valued in our contemporary society [5-7]. This crisis exacerbated existing and deeply rooted inequalities such as underfinancing in the care sector and the chronic shortage of caregivers (both in the health and social sector) [8]. However, contrary to the negative narrative, increased longevity and good health allow older adults to meaningfully contribute socially and economically, and maximize their well-being late into life [9-11]. To facilitate healthy and successful aging, the fast-growing digital technology, with all its drawbacks, barriers, and challenges, offers a staggering promise and opportunity [12].

Despite substantial mixed and inconclusive findings, several studies and reviews have demonstrated the positive impact of digital technologies on different dimensions of an older person’s life, including health, housing, services and transactions, mobility and transportation, access to information, communication and work, recreation, and self-fulfillment [13-15]. Moreover, digital technologies play a substantial role in improving older people’s quality of life and independence [16-18]. However, a review reported an ambivalence toward digital technology due to negative effects such as a sense of privacy and personal security breaches. Whereas, personal safety during emergencies was reported as a positive effect of owning a mobile phone [18].

Over the past decades, digital technology use among older populations has grown exponentially both in the mainstream (day-to-day lives) and assisted care (health and social care) [19,20]. Changes in the workplace and the “digital by default” strategy for delivering public services are among contributing factors *forcing* older people to engage digitally [21]. Digital engagement in health promotion and social support through health information is also growing. However, the breadth and the extent of digital technology use among older people remains limited to communications such as sending or receiving emails, instant messaging, video calls (Skype), and making voice calls [14]. A perceived or actual lack of interest, skill gaps, and socioeconomic factors were mentioned as possible reasons for the limited use of digital technologies [14]. Besides, the age-related decline in vision, hearing, cognition, and dexterity also attribute to the limited use of digital technologies [22-24].

Comparatively, there is a discrepancy in digital involvement, access, and connectivity between the younger and older populations [16,24]. For instance, in the United Kingdom between 2014 and 2019, a significant proportion of the older population never connected digitally at all or had not used the internet over the past 3 months. The 2019 Office for National Statistics (ONS) survey showed 13.5% of older people aged 65 to 74 years old and 47% of those 75 years and older never used the internet [16]. A similar population-based study in 7 European countries reported only 12% internet use among older people (60 years and older), of whom 64% used it for health-related issues [25]. In the United States, smartphone ownership among older people 65 years and older is significantly lower in comparison to the national average (81%; ie, 59% of those between the ages of 65 and 74 years are smartphone owners, but it falls to 40% among those 75 years and older) [26].

To create a digitally inclusive and accessible world, the International Organization for Standardization recommends human-centered and accessible designs (ISO 9241-11:2018) [27]. Adaptation guidelines such as text font size, screen setting, contrast, and color adjustments are among the recommended standards. These modalities enable older people with physical disabilities to engage digitally [28]. However, technology designs are mostly driven by technology push rather than user demand pull factors. Additionally, the fast-evolving nature of digital technology makes it challenging for older people to catch up and sustain engagement with the adaptation guidelines.

### Digital Engagement Later in Life

To thrive in the increasingly digitalized world, an acquaintance with technology is inadvertently becoming a mandatory way of life [21]. Despite the current assumption that older people are not using digital technologies, many studies have indicated that older people are competent and skilled digital technology users [29,30]. Still, there is a gap in evidence, with some key questions that require illumination:

1. What are the contributing factors to the digital inequality between the age groups?
2. How can we understand older people’s digital technology use?
3. What constitutes the diversity of digital technology use?

The term “digital engagement or disengagement” has been widely used in marketing research with an aim toward promoting marketing strategies to end consumers [31-33]. Factors like brand factor, product factor, consumer factors, and content factors have been the main focus of these studies [34]. Though the factors are intertwined, this review will focus on studies that explore drivers of technological nonuse, initial adoption, and sustained digital engagement from older people’s perspectives.

Overall, we propose to understand the current state of knowledge about older people’s digital engagement through the stages of digital engagement (nonuse, initial adoption, and sustained engagement). This will facilitate an ongoing drive to reduce digital inequality and, in doing so, provide new understandings to promote the well-being of older people. It will also help identify potential alternatives for older people who remain nonusers of digital technology.

### Digital Engagement Dimensions

**Figure 1:**
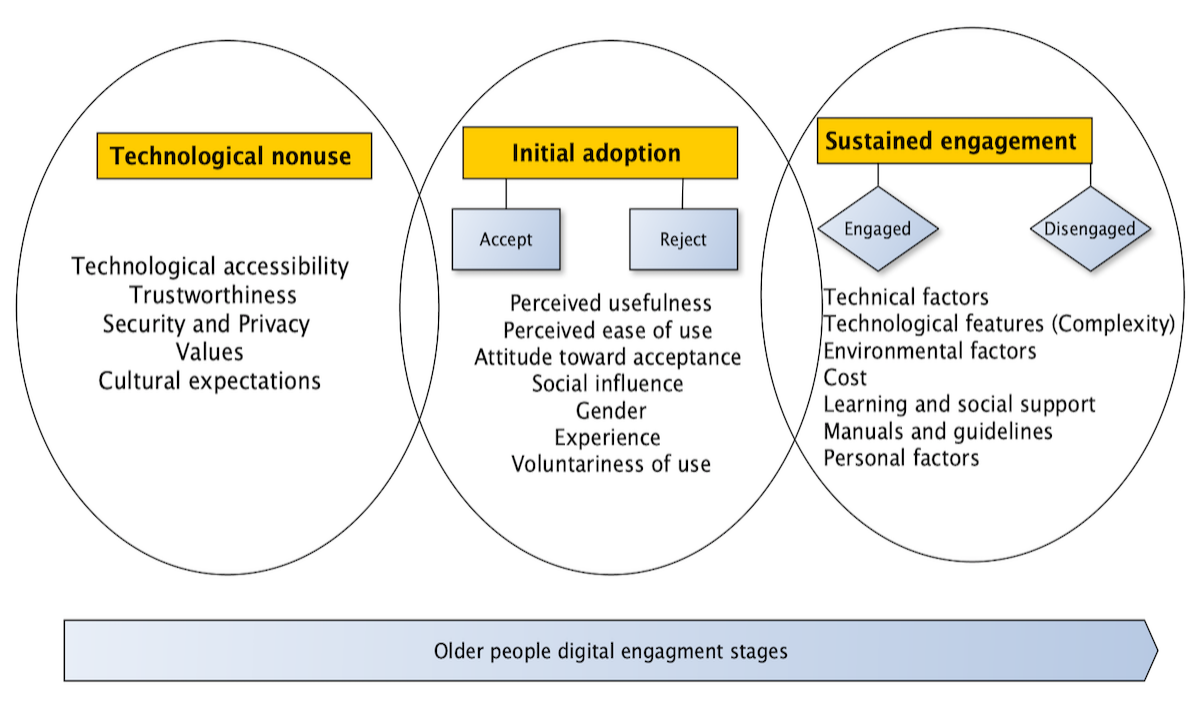
Older people’s digital engagement dimensions and stages later in life.

To facilitate this review, operationalizing older people’s digital engagement and disengagement is considered an important step in deciphering the continuum (Figure 1). This continuum with a three-stage approach involves technological nonuse, initial adoption or acceptance, and sustained digital engagement. This categorization will enrich the evidence mapping and the identification of barriers and facilitators for each dimension (initial adoption, sustained engagement, and technology nonuse). The description for each digital engagement dimension is provided in the following sections.

#### Stage I: Digital Technology Nonuse

Technological nonuse is not absolute as the term may suggest and goes beyond the absence of technology [35]. It is also a mistake to assume a person has not used a single digital technology, as use or nonuse is a constant negotiation and renegotiation to engage or disengage with technology. This also includes older people who access digital technology through their existing social support system (family and friends). To understand the possible factors affecting older people's engagement and disengagement, efforts to investigate the technological nonuse should be encouraged [35,36]. This will pave the way to understanding the bigger picture of digital exclusion among older people.

Governments across Europe (eg, the United Kingdom, Sweden, and Spain) have shown commitments to provide digital technologies through a framework (eg, universal service obligations for broadband) and accessible internet to citizens [37-39]. However, evidence has indicated that technological nonuse, later in life digital disengagement, and lower use rates are the main features of digital inequality among the older population [21,40]. The nonuse might involve technology, a service, an application, a platform, a communication medium, a set of practices, or some combination thereof. For example, the 2019 ONS survey in the United Kingdom showed 13.5% of older people aged 65 to 74 years and 47% of those 75 years and older never used the internet [16].

The drivers of technological nonuse are not only limited to sociodemographic and economic characteristics, but also the absence of tailored instructions and guidance, a lack of knowledge and confidence, and health-related barriers and costs [41]. According to Knowles and Hanson [17], accessibility and trustworthiness of the digital technologies, values, and religious and cultural expectations are salient determinants for older people’s technological nonuse. Moreover, complexity, security, and privacy issues also contribute to the technological nonuse among this age group.

#### Stage II: Initial Adoption

Studies dealing with user (older person) decisions to accept or reject digital technology and the drivers that influence the user decision will inform this stage. This will answer questions such as, “What influences users’ decision to use a particular digital technology?”

A considerable range of models and theories such as the Theory of Reasoned Action (TRA) [42], the Technological Acceptance Model (TAM) [43], the Unified Theory of Acceptance and Use of Technology (UTAUT) [44], the Diffusion of Innovations Theory [45], and Igbaria’s model [46] have been developed to facilitate an understanding of the drivers toward the favorableness and the unfavorableness of technology initial adoption [47]. The TAM [43,48] and UTAUT [44], a derivative of TRA, are among the prevailing theories. The TAM developed antecedent factors such as perceived usefulness, perceived ease of use, and attitude toward technological acceptance. Whereas, UTAUT, which is the extension of TAM, further developed the model by adding social influence and other moderating factors such as gender, age, experience, and voluntariness of use [44]. This review will scope studies that address these factors with age (65 years and older) as an important moderating factor [44]. Furthermore, this review will include qualitative accounts from older people’s perspectives, unlike the TAM and UTAUT models, which are widely used to quantify acceptance [49].

#### Stage III: Sustained Digital Engagement

People who actively used technology start to disengage due to age or the generational effects of aging [30]. According to Damodaran et al [50], sustained digital engagement is affected by the complexity and fast-changing nature of digital technology. Additionally, user’s low awareness about the availability of design adaptation modalities such as font size, color, and screen determined its sustained use. The manuals and guidelines on this design adaptation, which enhance older people’s capacity to adapt to technologies, are frequently inaccessible and outdated. Learning and support from existing social support such as family play a crucial role at this stage [50]. A similar study reported that sustained mobile technology use among older people was influenced by personal factors (physical, cognitive, and mental changes), environmental factors (financial costs, social influence, and learning to use technology), and technical factors (complexity and usability, absence of feedback, and design challenge) [29].

Sustained use is vital in understanding the digital divide among different socioeconomic groups [50]. However, studies suggested that it is one of the underresearched areas of digital engagement. A growing body of evidence has focused on understanding the early adoption, with the assumption that once people subscribe to the technology, they will keep using it. However, there is evidence that there will be a digital engagement negotiation and renegotiation between use and nonuse and vice versa [21]. Therefore, this review will include studies focused on factors that prevent or promote sustained use among older people.

### Scoping Review Rationale

There is a growing body of literature that often gives glowing reviews on the positive effect of digital technology engagement among older people. However, there is a gap in comprehensive reviews of evidence understanding the complexities of the barriers and facilitators of older people’s digital engagement. This review will summarize the current state of knowledge concerning older people’s perspectives on digital engagement and disengagement from technological nonuse, initial adoption, and sustained use. In addition, the varieties of technologies used or being used in social and health care for older people will be identified.

Studies have shown that the use of digital technology will have a great impact on different dimensions of older people’s lives, for example, quality of life [18], decision making [29], and mobility and social connectedness [14]. However, there are no reviews of existing studies that summarized the state of knowledge from older people’s perspectives, specifically the drivers of engagement and disengagement from technological nonuse to initial adoption and sustained use. This scoping review aims to provide a base for a more comprehensive understanding of digital engagement among older people. The findings will inform older people, designers, developers, and decision makers about practical implications. In addition, this review will set an agenda for future research and further in-depth understanding of older people’s digital engagement.

The findings from this review will inform the extent of evidence on older people’s digital engagement, inform the extent and the breadth of the knowledge about barriers and facilitators of older people’s digital engagement, and delineate the scope of what we already know. Further, these findings will indicate the gaps in the ongoing research of the issue.

### The Rationale in Light of the COVID-19 Pandemic

As of November 2020, there have been over 50 million COVID-19 cases and over 1 million deaths worldwide. Governments worldwide have implemented different levels of public health infrastructures such as lockdowns, social distancing, testing, contact tracing, and isolation measures [51]. As a result, digital technology use as a modality for coping with the crisis and socioeconomic continuity has substantially increased. For example, people are now using technology to work from home, to speak to their families and loved ones, and to source entertainment and information [52,53]. In addition, contact tracing apps were implemented in European countries, China, Singapore, and the United Kingdom [54,55]. Despite the unanticipated nature of the crisis and the higher vulnerability associated with age, the existing digital technology inequality among the age groups could imply low use or uptake of such services for the well-being of an older person, exacerbating the existing inequality [56]. In this new configuration of societal roles and innovative ways to tackle the transmission and stop the pandemic, future understanding of older people’s digital engagement will shed light on existing efforts to make technologies equitable.

## Methods

### Overview

The methodology for this scoping review is informed mainly by the Arksey and O’Malley framework for scoping reviews and will examine the extent, range, and breadth of evidence for the drivers of digital engagement among older people [57]. Additional recent methodological development on scoping reviews by Levac et al [58] and Tricco et al [59] (ie, PRISMA-ScR [Preferred Reporting Items for Systematic Reviews and Meta-Analyses Extension for Scoping Reviews] Checklist) will be incorporated in the main framework. The framework has 6 steps described in the following sections.

### Stage 1: Identifying Research Questions

Identifying relevant and broader research questions is the first step in the process of a scoping review. Our review questions are as follows:

1. What is known from the existing literature about the perspectives of older people on digital technology engagement?
2. What digital technologies have been used in the health and social care of older people?

### Stage 2: Identifying Relevant Studies

A comprehensive search of identified electronic databases will be conducted to locate relevant studies. Our search will include primary databases such as the Association for Computing Machinery Digital Library; Library, Information Science, and Technology Abstracts; MEDLINE; PsycINFO; CINAHL; and ScienceDirect. The search will be complemented by interdisciplinary (Web of Science, EBSCO, and Scopus) and secondary databases (Cochrane library and Joanna Briggs Institute [JBI] reviews). In addition, common grey literature sources from key journals (JMIR, the Journal of Gerontology, and the Journal of Gerontechnology) and Google Scholar will be included. Additional manual searches of peer-reviewed and grey source literature on the current COVID-19 crisis and the role of digital technology engagement among older people, published from December 2019 onward, will be included to support the review rationale.

Taking into consideration the research question and the JBI recommended population, concept, and context (PCC) approach, keywords and their synonyms, plurals, spellings, and acronyms will be used to develop a comprehensive search strategy as follows.

1. The population of this study is limited to studies conducted among older people 65 years and older. Terms such as “older person,” OR “older people,” OR “elderly,” OR “geriatric,” OR “old,” OR “frail,” OR “older user” will be used to form the population.
2. The concept will include studies dealing with digital engagement. Terms such as “digital,” OR “digital technology,” OR “digital engagement,” OR “digital technology engagement,” OR “technology” will form the concept.
3. There will be no restriction by context in terms of the geography of the studies.

All identified literature from our broad search strategy will be exported to the EndNote library manager and Evidence for Policy and Practice Information (EPPI) Reviewer 4 for the two-stage screening (title/abstract and full article).

### Stage 3: Study Selection

Inclusion and exclusion criteria to select studies will be generated based on the scope of the inquiry. Accordingly, we will use the iterative search strategy as we go back and forth to refine the search strategy and study selection.

#### Inclusion Criteria

Peer-reviewed articles will be the primary target, but also, grey literature sources with important insights into the scope will also be included to enrich the review. The inclusion criteria will be in line with the PCC of the studies described as follows:

1. Participants/Population: In this scoping review, we will include only literature that focused on digital technology among older people aged 65 years and older. Technological research with different age groups or a cross-age categorization will be excluded.
2. Concept/Condition: The primary concept in this study will be digital engagement. Studies that investigated digital engagement among older people and the determinants for technological nonuse, initial adoption, and sustained digital use will be included. In addition, studies that investigated different aspects of older people’s digital technology engagement, digital inclusion and divide, and other intersection features between old age and digital technology will be included.
3. Context: The context of this study will be global.

#### Study Identification

The study selection will involve two stages of screening. EPPI Reviewer software version 4 (from Evidence for Policy and Practice Information and Co-ordinating Centre) will be used to facilitate the screening process.

1. Title and abstract screening will be performed according to the inclusion and exclusion criteria.
2. Articles qualified by the title and abstract screening will be further considered for full article appraisal. Full articles will be accessed through the University of Brighton library, interuniversity library resources, and contacting the authors. The search results, screening process, and reasons for exclusion will be presented using the PRISMA-ScR flow diagram.

### Stage 4: Charting the Data

Important variables from studies found to be eligible for final inclusion in the scoping review will be extracted using a customized data extraction tool. The extracted variables will inform the scope and the breadth of the existing literature on older people’s digital engagement (Textbox 1). Variables such as study design, source of data, study size, study setting, study population, digital technology used, stage of digital engagement under study, and the barriers and facilitators of digital engagement among older people will be extracted.

**Variables to be extracted by review questions.**

- Authors
- Year of publication
- Aim of the research
- Research setting or place
- Methodology (study design, interventions, description, and analysis techniques)
- Outcomes
- Study population and sociodemographic characteristics (sample size, mean age, gender, and economic conditions)
- Digital technology under investigation (everyday and health technologies)
- Stage of digital engagement explored
- Barriers and facilitators identified
- Older people’s experience
- Research gaps and recommendations
- Keywords used for the study

### Stage 5: Collating, Summarizing, and Reporting the Results

An extension of the PRISMA-ScR flow diagram and guideline for reporting scoping reviews will be used to describe and collate the results of the final review [58,59]. The scoping will involve quantitative analysis (ie, frequency analysis), numerical description and common characterization of the studies by a study setting (geography or distribution), type of the study designs, the mean age of the study participants, and other features. Finally, the qualitative analysis will be conducted using the content analysis technique. Conceptual categories and definitions will be formed to inform the meanings, barriers, facilitators, and experiences of older people related to digital technology engagement. These categories will be used to generate themes. Levac et al [58] recommended qualitative content analysis to facilitate the summary and make sense of the extracted variables. This relational conceptual analysis will help explore relationships between the concepts extracted from the articles in the field. Charting of important variables and a narrative description of the findings will be presented in the review report.

### Stage 6: Consultation Exercise

We will conduct a consultation based on the identified preliminary literature findings on the topic of interest with identified stakeholders including advocacy groups, older people, academicians, digital developers, practitioners, and other early-stage researchers. This consultation exercise will be done after the preliminary electronic search on the common databases. The findings from the consultation exercise will inform our revision of the research question and refine the search strategy. The findings from the consultation exercise will be thematically presented.

### Dissemination and Ethical Requirements

We will comment on the ethical approval status of the included studies. However, for this review, ethical approval is not required since it uses publicly available sources. The key finding from this scoping review will be made available online and will be disseminated to key stakeholders.

## Results

We have conducted a preliminary search of the primary databases. We expect the final database search of this review to be completed in May 2021. We envisage disseminating the findings from this systematic scoping review in a scientific peer-reviewed journal.

## Discussion

We conceptualized older people’s digital engagement in a three-stage continuum from nonuse and initial adoption to sustained engagement. The findings from this review will identify the extent and nature of empirical evidence on how older people digitally engage and the associated barriers and facilitators at each stage of the continuum.

## Abbreviations

EPPI: Evidence for Policy and Practice Information
JBI: Joanna Briggs Institute
ONS: Office for National Statistics
PCC: population, concept, and context
PRISMA-ScR: Preferred Reporting Items for Systematic Reviews and Meta-Analyses Extension for Scoping Reviews
TAM: Technological Acceptance Model
TRA: Theory of Reasoned Action
UTAUT: Unified Theory of Acceptance and Use of Technology
